# Sarcoidosis of the spermatic cord – case report and literature review

**DOI:** 10.1186/s12610-022-00158-8

**Published:** 2022-05-19

**Authors:** Magdalena Ostrowska, Piotr Świniarski, Adam Ostrowski, Filip Ryszard Kowalski, Jan Adamowicz, Dariusz Grzanka, Tomasz Adam Drewa, Kajetan Juszczak

**Affiliations:** 1 Department of Urology, Nicolaus Copernicus University in Torun, Ludwik Rydygier Collegium Medicum in Bydgoszcz, Bydgoszcz, Poland; 2 Department of Clinical Pathomorphology, Faculty of Medicine, Nicolaus Copernicus University in Torun, Collegium Medicum in Bydgoszcz, Bydgoszcz, Poland

**Keywords:** Aarcoidosis, Granuloma, Spermatic cord, Scrotal mass, Case report, Sarcoïdose, Granulome, Cordon spermatique, Masse scrotale, Cas clinique

## Abstract

**Background:**

Sarcoidosis is a multi-system disease characterized by the formation of non-caseating granulomas in various organs. The lungs remain the most frequently affected organ, whereas lesions in the genitourinary system affect around 0.2% of patients. The primary site found in the spermatic cord is extremely rare.

**Case presentation:**

We present a patient’s case where the spermatic cord involvement was the first manifestation of sarcoidosis. For several months, a number of tests had been performed, which showed, among others, non-caseating granulomas in pathomorphological material, bilateral hilar lymphadenopathy, and leukopenia with lymphopenia. Tumor markers were normal. Infection with urogenital pathogens (including *Chlamydia Trachomatis*, Neisseria gonorrhea, *Mycoplasma hominis*) was excluded. The patient did not report any general symptoms such as fever, excessive fatigue, weight loss. He denied swelling, shortness of breath. At the same time, a complete differential diagnosis was carried out, and the extent of the disease was assessed. Due to interdisciplinary management, the patient’s quality of life and fertility is preserved. In the discussion, we present the diagnosis, treatment, and prognosis of such patients.

**Conclusion:**

Sarcoidosis is a multi-system disease, which should not be omitted in the differential diagnosis. Selective excision of the lesion with intraoperative examination plays a significant role while establishing a diagnosis. However, in the primary site in the genitourinary system, the diagnosis is challenging.

## Résumé

**Contexte:**

La sarcoïdose est une maladie multisystémique caractérisée par la formation de granulomes non caséeux dans divers organes. Les poumons restent l’organe le plus fréquemment touché, alors que les lésions du système génito-urinaire affectent environ 0,2% des patients. La découverte d’un site principal dans le cordon spermatique est extrêmement rare.

**Présentation du cas:**

Nous présentons le cas d’un patient où l’atteinte du cordon spermatique a été la première manifestation d’une sarcoïdose. Pendant plusieurs mois, un certain nombre de tests ont été effectués, qui montraient, entre autres, des granulomes non caséeux dans le matériel pathomorphologique, une lymphadénopathie hilaire bilatérale, et une leucopénie avec lymphopénie. Les marqueurs tumoraux étaient normaux. Une infection par des agents pathogènes urogénitaux (y compris *Chlamydia Trachomatis*, *Neisseria gonorrhea*, *Mycoplasma hominis*) a été exclue. Le patient n’a signalé aucun symptôme général tel que fièvre, fatigue excessive, ou perte de poids. Il a nié toute œdème ou essoufflement. Dans le même temps, un diagnostic différentiel complet a été effectué et l’étendue de la maladie a été évaluée. Grâce à la prise en charge interdisciplinaire, la qualité de vie et la fertilité du patient ont été préservées. Dans la discussion, nous présentons le diagnostic, le traitement et le pronostic de ces patients.

**Conclusion:**

La sarcoïdose est une maladie multisystémique, qui ne doit pas être omise dans le diagnostic différentiel. L’excision sélective de la lésion, avec examen peropératoire, joue un rôle important lors de l’établissement d’un diagnostic. Cependant, en cas de localisation du site primaire dans le système génito-urinaire, le diagnostic est difficile.

## Introduction

Sarcoidosis is a multi-system disease characterized by the formation of non-caseating granulomas in various organs. The lungs remain the most frequently affected organ, whereas lesions in the genitourinary system are found in less than 0.2% of patients [1].

Diagnostics can be challenging because sarcoidosis may resemble many infectious entities or mimic malignancies. With an inadequate diagnosis, the patient is burdened with unnecessary treatment or surgeries that might have a negative impact on future fertility and the quality of life [2].

We present a patient’s case in which the spermatic cord mass was the first manifestation of sarcoidosis. The aim of the article is to emphasize that sarcoidosis is a multi-system disease that should not be forgotten about in the differential diagnosis. The following article is prepared in accordance with the CARE reporting checklist.

## Case presentation

A 33-year-old patient was qualified for resection of the left spermatic cord tumor with a simultaneous intraoperative examination. Since October 2020, the man had begun to feel an enlarged mass in the left groin. Initially, antibiotic therapy was initiated, with no improvement. Within 2 months, the patient was consulted by several doctors. Tumor markers (Lactate Dehydrogenase, Human Chorionic Gonadotropin, Alpha-Fetoprotein) were normal. Infection with urogenital pathogens (including *Chlamydia Trachomatis*, Neisseria gonorrhea, *Mycoplasma hominis*) was excluded. The patient did not report any general symptoms such as fever, excessive fatigue, weight loss. He denied swelling, shortness of breath. Simultaneously with the mass in the groin, there was a skin lesion (a 4 cm spot) in the armpit area, which disappeared after 2 weeks.

**Fig. 1:**
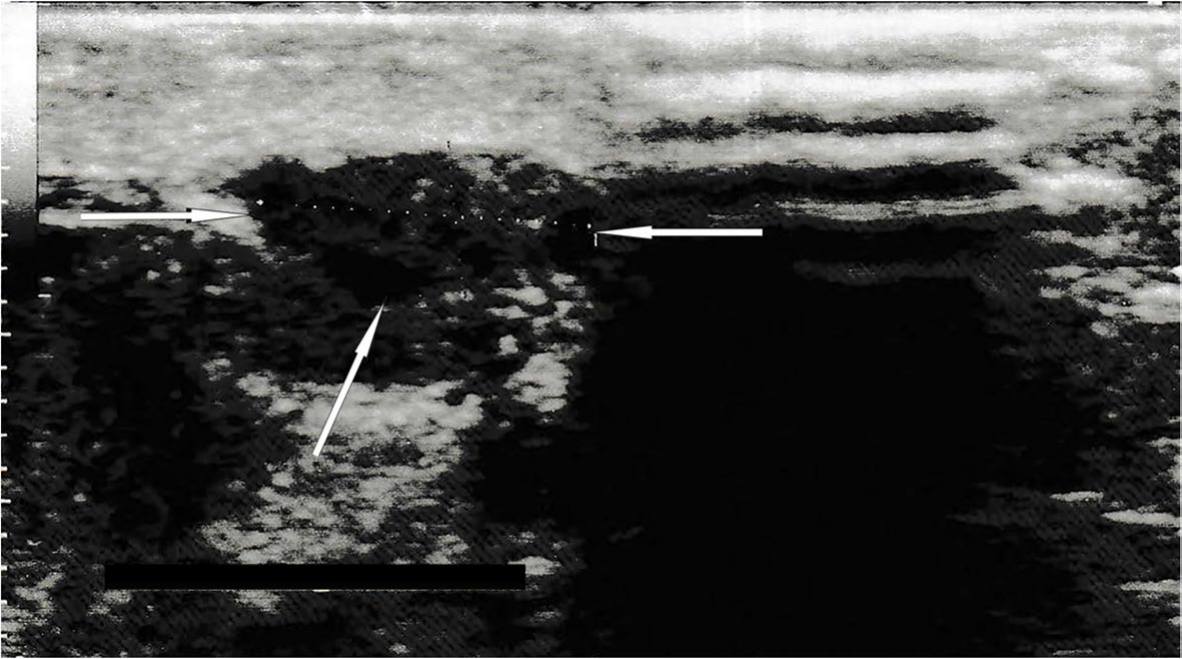
Ultrasound scan of the left side of the scrotum, showing small lesion with heterogeneous echogenicity

**Fig. 2:**
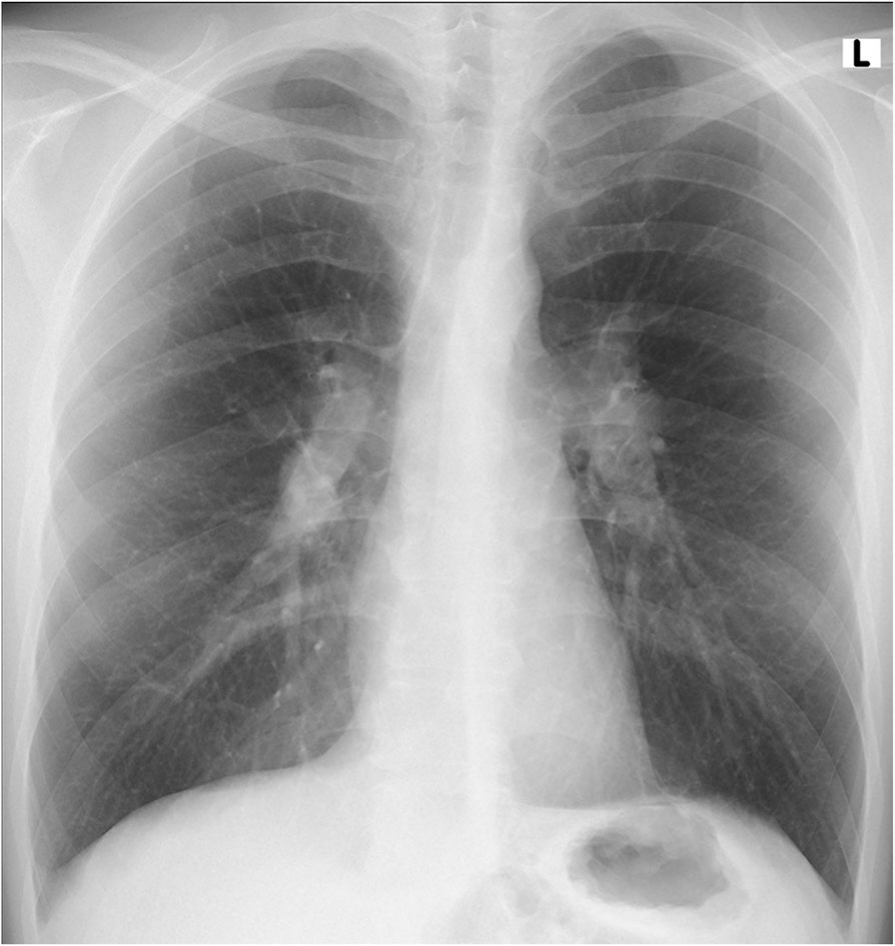
X-ray showing stage I sarcoidosis

Finally, the radiologist described the changes in the ultrasound as: “at the left epididymal body a 10x4mm tissue lesion of heterogeneous echogenicity; besides, epididymis and testicles without focal changes, right spermatic cord normal” (Fig. 1). In abdominal computed tomography (CT), the lesions were described as “asymmetrical thickening of the soft tissue structures of the left spermatic cord and left epididymis.”. The patient came with these results to our department, where we decided to excise the spermatic cord tumor with an intraoperative examination. If a neoplastic lesion had been diagnosed, inguinal orchidectomy would have been carried out. Due to the ambiguous nature of the lesion, the sperm was banked. Before surgery, a chest X-ray (Fig. 2) showed bilateral hilar lymphadenopathy, and sarcoidosis began to be suspected.

Laboratory abnormalities included leukopenia (White Blood Cells = 2.67 × 10^3^/ml; reference range: 4.5 to 11.0 × 10^3^/ml) and lymphocytopenia (0.69 × 10^3^/ml; reference range: 1.00 to 4.80 × 10^3^/m)l. Angiotensin-converting enzyme (ACE) was 90 ACEU (reference range: 20–70 ACEU). Among others, toxoplasmosis, echinococcosis, mononucleosis, human immunodeficiency virus, tuberculosis, and Candida infection were ruled out. For intraoperative examination, a tissue fragment of the left spermatic cord (1.8x1x0.5 cm) was sent, in which neoplasm was excluded, and granulomas were found. The lesion was removed selectively without affecting other structures of the spermatic cord.

**Fig. 3:**
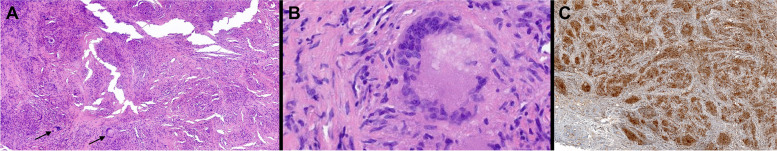
Pathological evaluation of removed lesion. **A** Non-caseating granulomas with tightly packed epithelioid cells, Langhans giant cells (arrows), and lymphocytes (hematoxylin and eosin stain = HE). Original magnification × 35; **B** Multinucleated giant cell (HE stain). Original magnification × 333; **C** CD68 staining highlights non-caseating granulomas. Original magnification × 18

In the further pathomorphological diagnostics, the histochemical staining of PAS (Periodic acid–Schiff), Ziehl-Nelsen, and the immunohistochemical staining of Cluster of Differentiation 68 (CD68) were used. It demonstrated: “numerous granulomas with Langhans type multinuclear giant cells; exclusion of tuberculosis mycobacteria” (Fig. 3). The description states that the morphological picture corresponds to changes in the course of sarcoidosis.

The patient was referred for further internal diagnostics. Moreover, Chest CT examination confirmed bilateral hilar lymphadenopathy (lymph nodes up to 14 mm). Hilar and mediastinal lymphadenopathy with no visible lesions in the lung parenchyma corresponded to stage 1 of sarcoidosis. The diagnostics of other organs, including the heart and eyes, was recommended, and no deviations were found. Therefore, together with the patient, the pulmonologist decided not to implement systemic treatment. Currently, the patient undergoes radiological check-ups every 2 months (ultrasound). Thus far, no disturbing lesions have been found. Laboratory tests show a gradual return of parameters to the reference range. In the latest blood tests, leukocytes and lymphocytes were at the lower limit of normal values. The patient claims his quality of life is entirely satisfactory.

## Discussion

Sarcoidosis manifestation in the genitourinary system is extremely rare and is estimated to affect less than 0.2% of patients [1]. In the largest review of 60 cases of sarcoidosis involving the male reproductive system, it was shown that epididymal lesions were found in 73% of patients, in testicles in 47%, in the spermatic cord in 8%, and the prostate in 3% [3]. As in our case, the vast majority of sarcoidosis appears between 25 and 40 years of age. The fact that sarcoidosis can have numerous manifestations and involve a number of organs results in a more demanding diagnostic process and management. A substantial number of patients require more than four doctor’s appointments for a diagnosis to be confirmed [4]. In the latest recommendations of the American Association for Thoracic Surgery, it is stated that the three main criteria for diagnosis include: a typical clinical picture, histopathological confirmation of noncaseating granulomas in at least one tissue, and the exclusion of other diseases with similar symptoms and course. However, experts emphasize that the diagnosis is never secure [5].

In the diagnosis of scrotal mass, ultrasound is the principal imaging modality. It is characterized by high resolution and can easily identify the lesion itself. However, it is impossible to make a differential diagnosis based only on ultrasound. The typical ultrasound presentation of sarcoidosis is a well-demarcated, hypoechoic, hypovascular nodule.

The major differential diagnoses of scrotal mass should take into consideration hernia, testicular tumor, lymphoma, liposarcoma, abscess, tuberculosis, and syphilis. Infection causes and lymphoma should be excluded first. Tuberculosis of the genitourinary system accounts for only 2 to 4% of tuberculosis cases, but the incidence has been increasing worldwide recently; therefore, it should also be considered [6]. The ultrasound examination can easily exclude hernia. Additionally, Magnetic Resonance Imaging (MRI) may be offered to the patient, but data on its use is also limited. MRI findings may include a medium signal in the T1-weighted image and a slightly increased T2-weighted image. Such findings are unspecific, as they might resemble inflammations, malignancies, and sarcoidosis [3]. In addition, MRI may help in deciding on the extent of excision. However, both imaging modalities are often insufficient in discrimination between malignant and benign lesions [3].

In the presented case, the first symptom of sarcoidosis was a palpable mass in the left groin. Considering the importance of oncological vigilance in the case of unilateral, palpable paratesticular mass, the initial diagnosis of spermatic cord tumor (SCT) was considered. Rodriguez et al. [7] note that it is often impossible to distinguish neoplasms of the epididymis from benign conditions. Therefore, the standard of treatment for SCT, 25% of which are potentially life-threatening malignant tumors, is radical orchiectomy. The most common form is orchiectomy, with high cord ligation, and wide excision of surrounding soft tissues structures within the inguinal canal [7, 8].

As in our case, sarcoidosis of the genitalia is usually described as a painless mass [9–11]. Nevertheless, in the case of the compression of the nerves, pain or tenderness could be expected. In such a case, surgery would be advised. In addition, although extremely rare, bilateral compression of the spermatic cord vessels could lead to azoospermia [12, 13]. In such a case, data on treatment modalities are confusing [12]. Corticosteroids could be an option instead of surgery [13]. In the case of oligospermia, the patient should be offered sperm banking.

We found no data on the risk of deterioration of sperm quality after selective lesion removal. On the other hand, ligation techniques of the spermatic cord varicocele veins are well described. The microsurgical technique was superior to open techniques, with only 0.44% of hydrocele formation vs. 7.30–8.24% in the latest [14]. Damage to the spermatic cord arteries and lymphatics during surgery may lead to atrophy and necrosis of the testis and hydrocele formation [15]. We can suspect that the same mechanism applies in removing the sarcoidosis of the spermatic cord. Therefore, microsurgical techniques are advised.

Furthermore, one-sided orchiectomy still does not compromise the paternity rate. In a large study in Norway, among men who underwent one-sided orchiectomy due to testicular cancer without further treatment, 92% of men had children without the use of cryopreserved semen [16]. As long as sarcoidosis is one-sided and the other testis appears normal, the paternity rate should not be compromised. The percentage stays in line with global paternity levels [17].

One-sided orchiectomy should not compromise testosterone levels. On the contrary, even without the involvement of the genitourinary tract, patients with sarcoidosis often suffer from hypogonadism. Spruit et Al. [18] reported that 46.7% of patients had testosterone levels lower than 300 ng/dl. Hypofunctioning of the hypothalamus–pituitary-gonadal axis was suspected of causing hypogonadism. Azoospermia, teratozoospermia, and oligospermia are sometimes observed in sarcoidosis patients, but conservative treatment could be helpful in some patients [19].

Considering all the indications for the orchiectomy, suspicion of cancer remains the most important. Not only the lesion in the testis can resemble cancer, but pulmonary nodule and mediastinal lymphadenopathy can also resemble metastasis [20]. Therefore, sole removal of the tumor without orchiectomy remains an option in highly motivated patients whose cancer risk is small. The frozen section can help in the decision of the surgery extent. In the primary site of the granuloma in the scrotum, surgery can confirm sarcoidosis. If the sarcoidosis is already confirmed, surgery should also be considered if cancer is suspected, local symptoms occur, or in case of decreased sperm quality. The association between sarcoidosis and testicular cancer is described in the literature. The risk of testicular cancer is approximately 100-fold higher in patients with sarcoidosis in comparison to the general population [21, 22]. The pathomechanism is unclear, and some authors suggest granulomas to be a sarcoid-like reaction against tumor antigens rather than authentic sarcoidosis [23]. Still, close follow-up should be carried out even in secondary sarcoidosis, and in case of any doubt, surgery should be offered.

The chest X-ray image turned out to be the key in our case. Isolated bilateral hilar or mediastinal lymphadenopathy is described as stage 1. Although Scadding’s classification has referred to for over 60 years and has a poor inter-observer agreement, Kirkil et al. [24] justify its use nowadays, confirming in their study a good correlation with the assessment of prognosis and the risk of death. Laboratory blood tests may play a role as well. Karadag et al. [25] summarized that the abnormalities might include: anemia, leukopenia with lymphopenia, hypercalcemia, elevated levels of liver enzymes, hypergammaglobulinemia, and high C-reactive protein. Elevated ACE levels are not specific nor sensitive and are only observed in 60% of patients. Our patient’s cumulative history and examination results could suggest sarcoidosis but remained nonspecific. The hallmark for the diagnosis was the non-caseating granulomas described in our patient’s pathomorphological material from the spermatic cord [26].

As the disease presentation may vary from a mild condition, with almost no clinical repercussion, to severe involvement of many organs, the treatment strategy should be considered individually. Therefore, after exclusion of other critical organs involvement, we decided with the patient on a close follow-up without further interventions and pharmacotherapy. When treatment is indicated, it is usually based on corticosteroids, but other options like methotrexate, azathioprine, anti-tumor necrosis factor therapies are also used.

In most patients, the disease resolves spontaneously or under treatment within 2 years, and the prognosis based on limited data depends on the general stage of the disease [23]. Testicular cancer treatment is the most effective in early-stage. Therefore, if cancer is suspected, the surgery should not be deferred. However, after spontaneous remission, sarcoidosis reoccurs in only 3.7% of patients [26].

## Conclusions

In the case of an atypical scrotal mass, sarcoidosis should be considered. Selective excision of the lesion with intraoperative examination might be helpful in diagnosis. In the case of sarcoidosis with a primary site in the genitourinary system, the diagnosis is challenging.

## Data Availability

Not applicable.

## Abbreviations

ACE: Angiotensin-converting enzyme
CD68: Cluster of Differentiation 68
CT: Computed Tomography
HE: Hematoxylin And Eosin
MRI: Magnetic Resonance Imaging
PAS: Periodic Acid-Schiff
SCT: Spermatic Cord Tumor
